# Sequencing of a Patient with Balanced Chromosome Abnormalities and Neurodevelopmental Disease Identifies Disruption of Multiple High Risk Loci by Structural Variation

**DOI:** 10.1371/journal.pone.0090894

**Published:** 2014-03-13

**Authors:** Jonathon Blake, Andrew Riddell, Susanne Theiss, Alexis Perez Gonzalez, Bettina Haase, Anna Jauch, Johannes W. G. Janssen, David Ibberson, Dinko Pavlinic, Ute Moog, Vladimir Benes, Heiko Runz

**Affiliations:** 1 Genomics Core Facility, EMBL Heidelberg, Heidelberg, Germany; 2 Flow Cytometry Core Facility, EMBL Heidelberg, Heidelberg, Germany; 3 Institute of Human Genetics, University of Heidelberg, Heidelberg, Germany; 4 CellNetworks Sequencing Core Facility, University of Heidelberg, Heidelberg, Germany; 5 Molecular Medicine Partnership Unit (MMPU), University of Heidelberg/EMBL, Heidelberg, Germany; University of Bonn, Institute of Experimental Hematology and Transfusion Medicine, Germany

## Abstract

Balanced chromosome abnormalities (BCAs) occur at a high frequency in healthy and diseased individuals, but cost-efficient strategies to identify BCAs and evaluate whether they contribute to a phenotype have not yet become widespread. Here we apply genome-wide mate-pair library sequencing to characterize structural variation in a patient with unclear neurodevelopmental disease (NDD) and complex *de novo* BCAs at the karyotype level. Nucleotide-level characterization of the clinically described BCA breakpoints revealed disruption of at least three NDD candidate genes (*LINC00299*, *NUP205*, *PSMD14*) that gave rise to abnormal mRNAs and could be assumed as disease-causing. However, unbiased genome-wide analysis of the sequencing data for cryptic structural variation was key to reveal an additional submicroscopic inversion that truncates the schizophrenia- and bipolar disorder-associated brain transcription factor *ZNF804A* as an equally likely NDD-driving gene. Deep sequencing of fluorescent-sorted wild-type and derivative chromosomes confirmed the clinically undetected BCA. Moreover, deep sequencing further validated a high accuracy of mate-pair library sequencing to detect structural variants larger than 10 kB, proposing that this approach is powerful for clinical-grade genome-wide structural variant detection. Our study supports previous evidence for a role of *ZNF804A* in NDD and highlights the need for a more comprehensive assessment of structural variation in karyotypically abnormal individuals and patients with neurocognitive disease to avoid diagnostic deception.

## Introduction

Structural variants (SVs) including copy-number variants (CNVs), inversions and translocations are a major contributor to human genetic variation and neurodevelopmental disease (NDD). [1] Among the most frequent SVs are *de novo* balanced chromosome rearrangements (BCAs) that occur in approximately 0.2% of all newborns and in most cases are unrelated to clinical phenotypes. [2]–[3] However, *de novo* BCA carriers show an about 2-fold increased risk to develop intellectual disability, multiple congenital anomalies, and autism spectrum disorders as in many instances BCAs disrupt genes with important roles in neurodevelopment and brain function. [4]–[5] With this, BCAs pose a particular challenge to prenatal genetic counselling [6] and diagnostics of NDD.

It is generally assumed that the contribution of BCAs to neurocognitive disease could prove to be considerably higher if routine clinical procedures allowed for an easy BCA detection. Yet, BCAs are typically identified by laborious low-resolution methods such as karyotyping and fluorescence *in situ* hybridization (FISH). Recently, mate-pair library sequencing has been introduced as a powerful approach to characterize the breakpoints of clinically-identified BCAs at nucleotide resolution or query the genome for submicroscopic SVs. [5]–[10] Genome-wide mate-pair library sequencing relies on the joining and capture of distant sequences on the identical DNA-strand, followed by paired-end sequencing of the joined chimeric fragments. The resulting high spanning coverage of the entire genome enables SV detection with a high sensitivity and at moderate sequencing costs. [9] Importantly, application of genome-wide mate-pair library sequencing to individuals with BCAs and NDD revealed a previously unknown complexity of chromosome rearrangements in the vicinity of the breakpoints and beyond. [5], [10] This suggests that in some patients disruption of genes outside the clinically described BCAs could contribute to their respective neurodevelopmental phenotype.

Here we describe a patient where complex BCAs disrupt at least six genes, several of which are candidates for NDD. Of these, we show that the brain transcription factor and likely disease-relevant gene *ZNF804A* resides in a cryptic inversion that was beneath the resolution of routine clinical analyses and was only identified by sequencing. Our study demonstrates the power of genome-wide mate-pair library sequencing to derive reliable catalogues of clinically undetected SVs. It further highlights the need for a more comprehensive assessment of structural variation in individuals with chromosome aberrations and/or neurocognitive disorders to avoid diagnostic deception.

## Results

**Figure 1:**
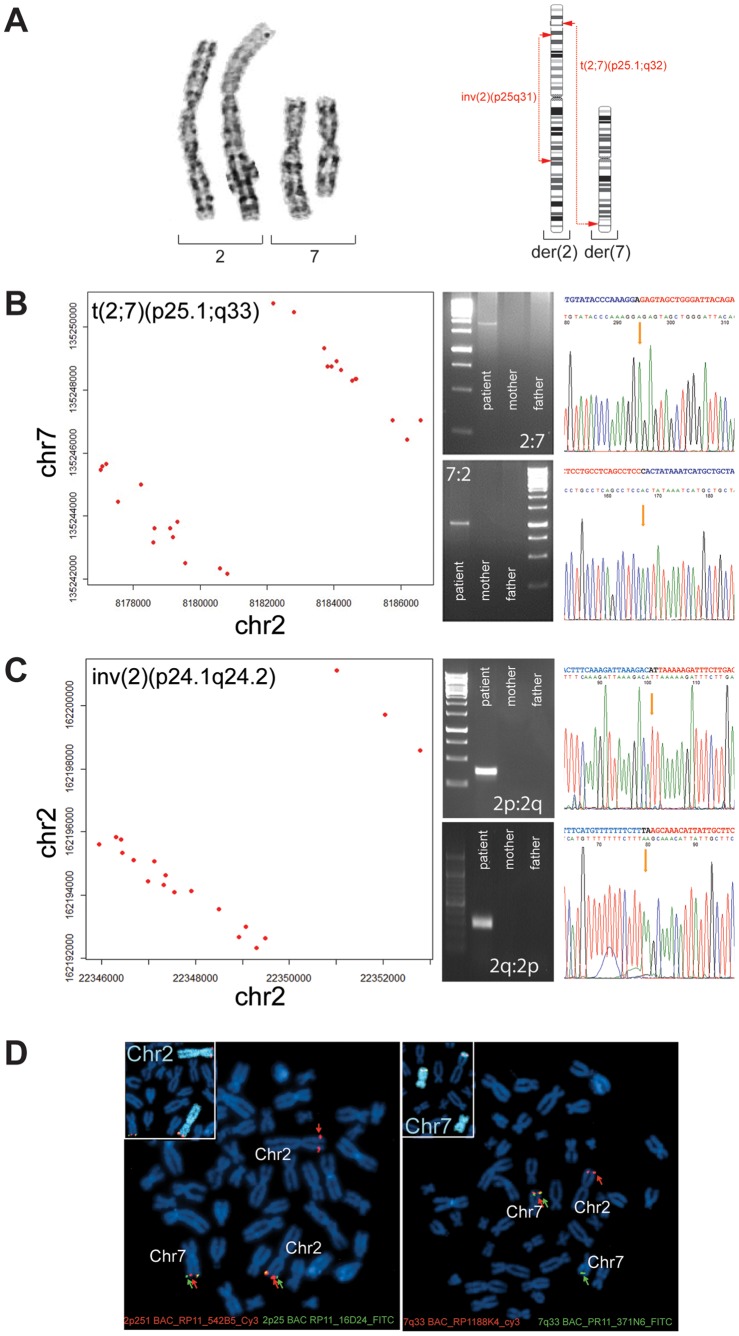
Base-pair level characterization of clinically-identified *de novo* balanced chromosome abnormality (BCAs). (A) Chromosomes 2 and 7 of the patient as visualized by GTG-banding. Breakpoint positions of BCAs reported from clinical analyses at karyotype level are indicated in red. (B,C) Graphical representation of anomalous-read (red dots) fusion positions for t(2;7) (B) and the paracentric inv(2) (C). Based on mate-pair library sequencing-identified gap positions primers were designed to amplify and validate anomalous regions by PCR and capillary sequencing at base-pair level. (D) Validation of re-annotated t(2;7) breakpoint positions by FISH with BAC-probes binding immediately adjacent to re-defined translocation sites.

We applied genome-wide mate-pair library sequencing to characterize structural variation in a male patient with neurodevelopmental disabilities and apparently balanced *de novo* chromosomal rearrangements (see Methods for clinical details). Karyotyping of chromosomes isolated from the patient’s blood lymphocytes identified two major *de novo* and apparently balanced chromosome rearrangements: a reciprocal translocation between chromosomes 2 and 7 involving bands p25.1 and q32 respectively; and a large pericentric inversion on the derivative chromosome 2 [der(2)] (**Figure 1A**), without signs of additional numerical or structural aberrations [46,XY,t(2;7)(p25.1;q32)inv(2)(p25q31)dn] (**Figure S1**). Comparative genome-hybridization analysis using Affymetrix 6.0 SNP-arrays (Affymetrix, Santa Clara, CA, USA) excluded CNVs larger than 100 kB, suggesting that the chromosome rearrangements observed at karyotypic resolution were apparently balanced.

In order to evaluate disease-relevance of these SVs we mapped the breakpoints at higher resolution using genome-wide mate-pair library sequencing [7], [9]. Patient recruitment protocols were approved by the institutional review board of Heidelberg University and the family’s informed consent was obtained. Genomic DNA of the patient was captured using Illumina 5 kb mate-pair sample prep kits according to the manufacturer’s instructions (Illumina, San Diego, CA, USA). In brief, distant sequences on the same DNA-strand were joined by circularization, and the purified joined fragments were paired-end sequenced on a single lane of Genome Analyzer IIx (Illumina). With median insert sizes of 5,012 bp this protocol generated 28.2 million read-pairs, resulting in a theoretical genome-wide spanning read-depth of 25.9-fold that enabled detection of SVs with a high sensitivity (see Methods for details on sequence analysis).

A total of 30 discordant reads allowed us to narrow the breakpoints of the reciprocal translocation t(2;7) to 1,393 bp on der(2) and 970 bp on der(7) (**Figure 1B**). PCR amplification of the chimeric regions followed by capillary sequencing validated the mate-pair data and revealed breakpoints at positions chr2∶8,181,790 and chr7∶135,245,984 (GRCh37/hg19). Both breakpoints carried the adenine at position chr2∶8,181,790 while the cytosine on chr7∶135,245,985 was lost. Apart from this 1 bp indel the rearrangements were balanced, with no signs of further SVs in the proximity of the breakpoints. The pericentric inversion inv(2) was successfully identified by 22 centromere-spanning discordant reads that localized the breakpoints within 4,703 bp on the p-arm and 3,490 bp on the q-arm of der(2) (**Figure 1C**). Capillary sequencing confirmed 1 bp insertions both at chr2∶162,196,595 and chr2∶22,350,265, with the adenines at the respective positions present at either side of the inversion. Again, no evidence was found for further rearrangements at or near the inversion sites. Overall, the pericentric inversion encompassed a genomic region of 139,846,330 bp.

Failure to amplify the chimeric fragments in DNA from the patient’s parents confirmed that both, the translocation as well as the pericentric inversion had occurred *de novo* (**Figure 1B,C**). As observed previously [5], sequencing considerably revised the clinically predicted karyotype (**Table 1**). FISH analysis with BAC-probes binding immediately adjacent to the newly-identified translocation breakpoints confirmed that the revised translocation sites were correct (**Figure 1D**). Taken together, even at a low sequencing read-depth as applied here, mate-pair library sequencing permitted us to unambiguously map four cytogenetically predicted breakpoints at a resolution high enough to correctly describe the exact nature of the underlying SVs.

**Table 1:** Genes disrupted by validated structural rearrangements (>10 kb) in the patient.

SV no.	revised karyotype#	breakpoint start	disrupted gene$	RefSeq transcript position	exon count	proposed functions	disease link	ref.	con-firmed	mRNA levels	fusion mRNA	brain expressed	patient&
1	**t(2;7)(p25.1;q33**)	chr7∶135,245,984	NUP205	chr7∶135,242,662–135,333,499	43	nuclear-cytoplasmic transport	Paget’s disease (assoc.)	[15]–[16]	yes	normal	yes	yes	1.9%
		chr2∶8,181,790	**LINC00299**	chr2∶8,347,273–8,468,549	8	unknown	NDD, neurodegeneration	[11]	yes	possibly increased		yes	n.r.
2	**inv(2)(p24.1q24.2)**	chr2∶22,350,265	AC068490.2	chr2∶22,156,208–22,753,977	5	unknown	n.r.	n.r.	yes	n.d.	yes	n.r.	n.r.
		chr2∶162,196,595	PSMD14	chr2∶162,164,786–162,268,228	12	protein degradation	2q24.2 syndrome (assoc.)	[12]–[13]	yes	normal		yes	1.8%
3	**inv(2)(q32.1q32.1)**	chr2∶185,679,985	**ZNF804A**	chr2∶185,463,093–185,804,214	4	transcription factor	NDD, schizophrenia/bipolar disorder (assoc.)	[5], [23]	yes	reduced	no	yes	38.7%
		chr2∶188,161,779	AC007319.1	chr2∶187,868,001–188,419,120	4	unknown	n.r.	n.r.	yes	n.d.		n.r.	n.r.
4	**del(19)(q13.4)**	chr19∶54,800,252	LILRA3	chr19∶54,799,855–54,804,221	7	immuno-receptor	prostate cancer/HDL-c (assoc.)	[30]–[31]	yes	n.d.	N/A	no/low	n.r.
		chr19∶54,809,071	N/A	N/A	N/A	N/A	N/A	N/A	yes	n.d.		N/A	N/A

# revision to clinical diagnostic reports in bold;

$ reported neurodevelopmental disease (NDD) genes in bold;

& acc. to Huang et al., 2010 [28].

**Figure 2:**
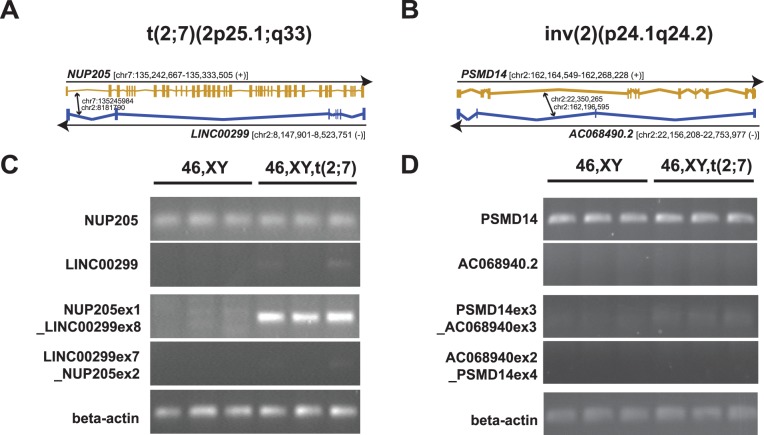
Nucleotide-level characterization of cytogenetically visible breakpoints identifies gene fusions that encode for abnormal transcripts. (A,B) Graphical representation of the four genes within the cytogenetically visible reciprocal translocation t(2;7)(2p25.1;q33) and the pericentric inversion inv(2)(p24.1q24.2) in which structural variants disrupt protein-coding gene regions in the patient. Sites of breakpoints are denoted by arrows. (C,D) To monitor whether predicted SV-induced fusion transcripts resulted in abnormal transcripts, total RNA from three biological replicates per proband was isolated from lymphoblasts of the patient (46,XY,t(2;7); lanes 4–6) and a healthy male control individual (46,XY; lanes 1–3). For each site of structural rearrangement mRNA-levels of both, the wildtype and/or pre−/post rearrangement transcript, as well as the predicted fusion transcript were amplified with target-specific primers by RT-PCR.

We next were interested whether the clinically-observed BCAs could explain the patient’s symptoms. Importantly, all four clinically predicted breakpoints disrupted annotated genes (**Table 1**). Specifically, chr2∶8,181,790 resides within intron7 of *LINC00299*, while chr7∶135,245,984 locates to intron1 of *NUP205*. Balanced reciprocal exchange at these positions suggested creation of two abnormal coding fusion products, one expressing exon1 of *NUP205* fused to exon8 of *LINC00299*, the other expressing exons1-7 of *LINC00299* fused to exons2-43 of *NUP205*. Indeed, mRNA of the NUP205ex1_LINC00299ex8 fusion was expressed in significant amounts in lymphoblasts (**Figure 2A**) and fibroblasts (not shown) of the patient, but not in cells of a healthy male control. Cellular levels of the combined wild-type and posttranslocation NUP205 transcripts (as amplified by primers targeting exons2-8) were not different from controls, while expression of wild-type and pretranslocation LINC00299 transcripts (as amplified by primers targeting exons2-6) appeared to be slightly increased. Consistently, quantitiative RT-PCR indicated 6.7-fold (relative to housekeeping gene beta-actin) to 9.4-fold (relative to RPL19) increased mRNA-levels of pre-translocation LINC00299 in patient relative to control cells (not shown). Similarly, the pericentric inversion resulted in fusion of exons1-3 of *PSMD14* with exons3-4 of *AC068490.2* (**Figure 2B**). In addition to this, the PSMD14ex3_AC068490.2ex3, but not the reciprocal fusion mRNA were observed at low levels in the patient’s cells, while PSMD14 wild-type and postinversion transcript levels (as amplified by primers targeting exons 4–5) remained unchanged.

Of the four disrupted genes, truncation of the brain-expressed large intergenic non-coding (linc) RNA LINC00299 was recently proposed as causative for neurodevelopmental disability of varying severity [11]. Notably, also in that study’s patient, wild-type and pretranslocation LINC00299 transcript levels were increased and some of the clinical symptoms – including impaired speech, coordination deficits, otitis media and oligohydramnios – overlapped with the patient described here (**Table 2**), suggesting disruption of *LINC00299* as potentially causative. However, also *PSMD14* and *NUP205* proved to be attractive candidate genes: The human deubiquitinase and constituent of the proteasome complex PSMD14 was previously found to be one of three candidate genes within a critical region on 2q24 where CNVs have been linked to intellectual disability [12] and - like multiple other genes associated with autism-like phenotypes – might have a role in proteasome-mediated synapse elimination. [13]–[14] Conversely, *NUP205* encodes for a soluble component of the nuclear pore complex (NPC) machinery that contributes to cargo selection during nuclear-cytoplasmic transport. Cells deficient for NUP205 fail to exclude nonnuclear macromolecules, amongst others vital transcription factors, from entering the nucleus [15] and exhibit an accelerated entry into mitosis, possibly due to local destabilization of NPCs facing centrosomes [16].

**Table 2:** Phenotypic comparison of the patient to previously described individuals with structural variants affecting *ZNF804A* or *LINC00299*.

symptom	patient	ZNF804A				LINC00299	
		Talkowski et al., 2012a [5]			DECIPHER CNV cases (n = 7)	Talkowski et al., 2012b [11]	
		DGAP180	father of DGAP180	CNV cases (n = 7)		DGAP162	CNV case L1
**gene disruption**	*ZNF804A* truncated; breakin *LINC00299* intron 7	complex chromosomal rearrangementstarting 229 kb downstream of 3′UTR		CNVs comprising multiple genes at 2q31.1–2q33.1	CNVs comprising multiple genes at 2q31.2–2q32.3	break in intron 6;exons 7–8 missing	deletion ofexons 7–8
**developmental delay**	yes	yes	yes	yes (n = 4)	yes (n = 7)	yes	yes
**cognitive impairment**	yes	yes	yes	yes (n = 7)	yes (n = 6)	yes	yes
**autistic/abnorm. behavior**	yes	yes	yes	yes (n = 1)	yes (n = 3)	yes	n.r.
**anxiety**	yes	n.r.	yes	n.r.	n.r.	n.r.	n.r.
**expressive speech impaired**	yes	yes	yes	yes (n = 1)	yes (n = 2)	yes	n.r.
**coord. deficits/ataxia**	yes	yes	n.r.	n.r.	yes (n = 1)	yes (progressive)	n.r.
**arachnoidal cysts**	yes (axial, tectum)	yes (axial, Sylvianfissure)	n.r.	n.r.	n.r.	no (partial agenesisof corpus callosum)	n.r.
**recurrent otits media**	yes	yes	n.r.	n.r.	n.r.	yes	n.r.
**dysmorphism**	no	no	no	yes (n = 3)	yes (n = 7)	no	no
**muscle hypotonia**	yes	no	n.r.	yes (n = 1)	n.r.	no	n.r.
**other**	oligohydramnios	IUGR, prematurity, pneumonia	tics	cleft palate,microcephalus, …	cleft palate, seizures, micrognathia,…	oligohydramnios, dilated fetal kidneys, seizures, disinhibition	seizures, treated for bipolar disorder
**family history**	consanguinity	family members with cognitive andspeech impairment, tics, anxiety		n.r.	n.r.	n.r.	n.r.

**Figure 3:**
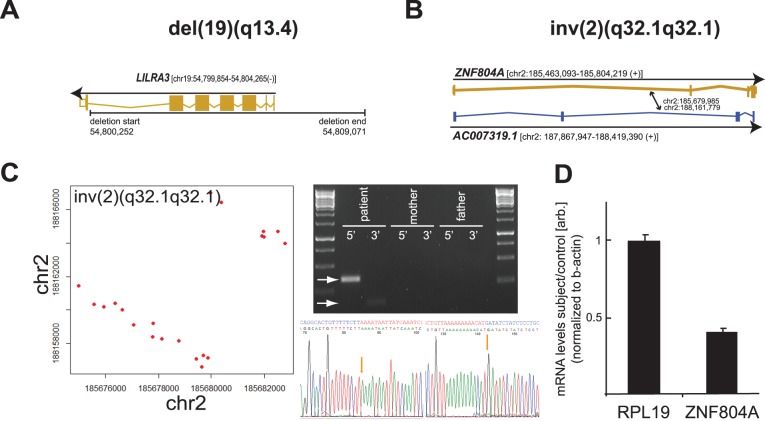
Genome-wide analysis for cryptic SVs identifies disruption of further neurodevelopmental disease candidate genes and demonstrates reduced expression of ZNF804A in patient cells. (A,B) Graphical representation of the three genes disrupted by an ∼10 kB deletion on chr 19 [del(19q13.4)] (A) and the cryptic paracentric inversion inv(2)(p32.1q32.1) in the patient. Sites of breakpoints are denoted by arrows. (C) Graphical representation of anomalous-read (red dots) fusion positions for the cryptic 2.49 Mb paracentric inversion on chromosome 2. Mate-pair library sequencing-predicted breakpoints 5′ and 3′ of the inversion were amplified with breakpoint-specific primers and validated at base-pair level by PCR and capillary sequencing. (D) mRNA-levels of ZNF804A and the housekeeping gene RPL19 were quantified by qRT-PCR from total RNA isolated from fibroblasts of the patient or a healthy male control and normalized to expression of beta-actin.

Conjoint disruption of at least three NDD candidate genes by apparently balanced SVs in one individual motivated us to investigate whether further genes in the patient’s genome could be disrupted by SVs. For this, we queried the mate-pair library sequencing data for “incidental” SVs below the cytogenetic resolution limit. As expected, the source data suggested multiple additional SVs of varying size in the patient’s genome. For instance, by setting a read-depth cut-off of six supporting discordant reads that aligned within 2x median library size (approximately 10 kb) of each other (see Methods), a total of 80 gene-affecting intrachromosomal rearrangements with >10 kb in size were called (**Table S1**). The overall 70 deletions and 10 inversions were located within or nearby a total of 129 annotated genes, of which 94 encode for proteins, 14 are untranslated transcripts, and 21 are pseudogenes. For the majority of these regions (n = 116; 89.9%) SV-boundaries could be reliably predicted. Of these, 112 were non-protein-coding, localized within intergenic regions or were confined to single introns, thus excluding disruption of coding elements. Apart from disruption of *NUP205* and *PSMD14*, a single deletion of ∼10 kb in size within the patient’s genome disrupted exons 1–6 of one allele of *LILRA3* (**Figure 3A**; **Table 1**). Most importantly, however, sequencing revealed a third major genomic rearrangement on chr2 below the cytogenetic resolution limit. Specifically, a cluster of 24 discordant reads suggested a paracentric inversion of 2.49 Mb on 2q32.1, with breakpoints residing within gaps of 396 bp and 2,947 bp. This previously undetected BCA also proved to be *de novo* and gene disrupting. Importantly, it fused two further genes: the processed transcript *AC007319.1* and the transcription factor *ZNF804A*. Position of both breakpoints within intron2 of *AC007319.1* and intron1 of *ZNF804A*, respectively, proposed significantly shorter or entirely absent gene products (**Figure 3B,C**; **Table 1**). Consistent with this, ZNF804A mRNA levels were reduced to 40% in the patient’s fibroblasts (**Figure 3D**). Due to the orientation of both genes no fusion mRNA was expected to result from the paracentric inversion.

Remarkably, several points of evidence suggest this cryptic paracentric inversion as at least equally likely to explain the patient’s phenotype than disruption of any of the NDD candidate genes within the cytogenetically visible BCAs: A recent study in a large cohort of individuals with NDD and autism spectrum disorders [5] identified two symptomatic carriers, father and son, of a reciprocal translocation that truncated *ZNF804A* 229 kb downstream of the end of its 3′-untranslated region. As with the patient described here, symptoms of these individuals included neurodevelopmental and behavioural deficits, ataxia, recurrent otitis media, and notably severe expressive speech delay and arachnoidal cysts (**Table 2**). With a frequency of <1% [17] and 2.6% [18], respectively, expressive speech delay and arachnoidal cysts are relatively rare in paediatric patients. Several further NDD individuals with CNVs at this locus have been reported as aphasic or showing severe speech impairment (**Table 2**) [19], indicating that ZNF804A might have a role in language acquisition or initiation. The gene encodes for a zinc-finger binding transcription factor that interacts with ataxin-1 [20] and regulates expression of genes involved in neurotransmitter signalling and cell adhesion, which proposes a reduction in ZNF804A as relevant for neuronal morphology and/or synaptic transmission. [21]–[22] Importantly, genome-wide association studies have identified ZNF804A as one of the most compelling loci associated with schizophrenia and bipolar disorder [23]–[25]. As carriers of the most strongly associated risk allele show increased ZNF804A expression [26], it has been hypothesized that altered levels of ZNF804A could cause pleiotropic effects, resulting in neuropsychiatric disease of variable manifestation [5], [27]. Our identification of the, to our knowledge, first indvidual with NDD where the coding sequence of one almost entire *ZNF804A* allele is specifically disrupted now strongly supports this assumption.

Consistent with previous knowledge [9] and known for the mate-pair sequencing protocol applied (that requires genome assembly from 36 bp reads) we considered that several of the SVs predicted from sequencing could be false-positives, and that deeper, more costly sequencing would be required to unambiguously demonstrate the presence or absence of SVs at a genome-wide scale. Therefore, to gain a more systematic insight into how accurately mate-pair sequencing describes submicroscopic structural variation in an NDD patient with complex BCAs, and also to validate the exact nature of the novel paracentric inversion, we subjected chr2 and der(2) to deep sequencing. For this, fluorescent-labelled chromosomes were separated from the patient and a male control individual’s lymphoblasts by flow-cytometry [8]. This allowed us to enrich both, chr2 and der(2) by 4.99- and 4.10-fold, respectively, over all other chromosomes, and to sequence the enriched fractions at a mean read-depth of 20.7 for chr2 and 18.7 for der(2) using a single lane of HiSeq2000 (Illumina) per chromosome fraction (**Figure S2**; for experimental details see Methods). Indeed, deep sequencing confirmed 10 of the 11 SVs larger than 10 kb predicted for chr2 from the mate-pair data, among them all 6 SVs within or nearby annotated genes (**Table S1**). Of these, the 2.49 Mb paracentric inversion, like the clinically described BCAs, localized to der(2), while the additional validated SVs evenly distributed on chr2 and der(2). The discrete, balanced nature of the chromosomal abnormalities were dissimilar to previously reported chromothripsis related complex rearrangements [10]. Therefore chromothripsis was considered unlikely as a possible reason for NDD in this patient. Taken together, the validation rate of 91% for SVs called on chromosome 2 and the high resolution of the breakpoint discovery and localization provide strong arguments that mate-pair sequencing as applied here has the potential to outcompete routine approaches for clinical-grade genome-wide SV detection.

## Discussion

In summary, our study has identified at least four NDD candidate genes in the patient’s genome that are disrupted by BCAs. Of these, a very compelling candidate, *ZNF804A*, locates to a cryptic rearrangement that had been missed by clinical procedures. The results of this study are noteworthy for four reasons: First, we demonstrate that genome-wide mate-pair library sequencing using an off-the-shelf enrichment kit is a powerful strategy to not only robustly characterize complex BCAs predicted from prior cytogenetic information; but also to discover cryptic SVs of probable relevance to a patient’s phenotype. Arguably, large-insert library sequencing is challenged by repetitive regions in the genome that interfere with correct alignment of the short discordant reads, resulting in high false-discovery rates [9]. Also, in relation to deep sequencing of the whole genome, the sensitivity to detect small insertions and deletions may be suboptimal using the mate-pair approach as libraries may be contaminated with non-mate paired reads still present after mate-pair enrichment. However, in this study only one of 11 SVs predicted for the studied patient’s chr2 and der(2) by mate-pair library sequencing failed to be validated by deep sequencing of flow-sorted chromosomes. Deep sequencing further confirmed that with stringent analysis settings and manual curation of the mate-pair sequencing data as applied here it is possible to almost eliminate falsely called intrachromosomal SVs larger than 10 kb. This strongly suggests that in a genome with an only moderate SV-burden as analyzed here, the specificity of mate-pair library sequencing for genome-wide *de novo* SV detection could be considerably higher than the 68% validation rate reported from a patient with chromosomes fragmented by chromothripsis [10]. In conclusion, the ability to identify and characterize multiple small SVs at near-to nucleotide resolution, the moderate costs, and the short turn-around time that enable reliable breakpoint characterization even in a prenatal diagnostic setting [6] predispose genome-wide mate-pair library sequencing as a versatile and robust analytical tool for next-generation cytogenetic diagnostics.

Second, our study confirms previous reports hinting at a surprising structural variability in BCA carriers [5], [9]–[10] and for the first time identifies a previously undetected “incidental” BCA as a very likely contributor to disease. Based on a certain phenotypic overlap with two previously reported individuals [11], restriction of our analyses to selected chromosomes and clinically described translocation breakpoints would have most likely resulted in imprecisely reporting disruption of the known NDD gene *LINC00299* as the most probable cause for the patient’s neurocognitive disorder. Instead, our data strongly argue that an individual undergoing diagnostic evaluation for NDD should be characterized for structural variation at a genome-wide level and as comprehensively as possible. This is strongly supported by a recent study where 12 of 36 NDD patients with clinically known BCAs showed unexpected additional chromosome rearrangements in the proximity or distant from the predicted breakpoints that clinical routines had failed to identify [5]. It will be interesting to further evaluate how such “incidental” SVs contribute to the respective clinical phenotypes, and if they could be one factor that drive the pronounced clinical variability of neurocognitive disease.

A third insight from our study is that SVs, and in particular *de novo* BCAs, should not be neglected as a cause for disease in individuals with an independently increased likelihood for inherited disorders. Due to consanguinity of the parents we expected a high degree of homozygosity in the studied patient. This is supported by a total of 154 homozygous coding missense variants on the patient’s chr2 and der(2), none of which, however, is likely to be deleterious (**Table S2**). While we cannot fully exclude a yet unknown autosomal-recessive contribution to the patient’s NDD phenotype elsewhere in his genome, it is interesting to note that none of the multiple SVs discovered here would likely have been identified by analyzing the patient’s exome. Mate-pair library sequencing thus could ideally complement exome profiling to more comprehensively assess variation in an individual’s genome and clarify the cause of disease in patients where exome sequencing fails.

Finally, the importance of systematically acquiring such data together with adequate phenotypic information is highlighted by the challenge to weigh the contribution of each of the four disrupted NDD candidate genes (*LINC00299*, *NUP205*, *PSMD14* and *ZNF804A*) to the patient’s phenotype. Huang et al. predicted the probability of haploinsufficiency for *ZNF804A* (38.7%) as considerably higher than for *NUP205* (1.9%) or *PSMD14* (1.8%) [28]. This, together with the patient’s phenotypic similarity to previously described individuals with impaired ZNF804A function [5], supports the assumption that monoallelic disruption of *ZNF804A* could be the predominant driver of symptom constellation in this patient. One possibility to further clarify this, which in this case was declined by the patient’s family, could be functional MRI, as adult carriers of the common ZNF804A schizophrenia risk allele show reduced cortical thickness and connectivity between and within the dorsolateral prefrontal cortex [29]. Alternatively to monogenic impediment of ZNF804A, the concerted loss-of-function of several SV-disrupted genes with relevant roles in neurodevelopment could generate a genomic disorder unique to the studied patient. While this is an attractive hypothesis that may well explain the pleiotropy seen in many NDDs, it will be almost impossible to further characterize such level of complexity in animal or cellular models. Instead, a concerted initiative to obtain high-resolution structural together with phenotypic information in large enough numbers of healthy and diseased individuals, as exemplified for coding variation [30], may help to not only distinguish damaging from neutral SVs, but reveal fascinating insights into brain function in health and disease.

## Materials and Methods

### Ethics Statement

The study and consent procedure was approved by the institutional review board of Heidelberg University Medical Faculty. The study protocol conformed to the ethical guidelines of the 1975 Declaration of Helsinki in its latest version. The parents provided written informed consent on behalf of their child to participate in this study and to publish potentially identifying information on the index case. Parents and the healthy control provided written informed consent for themselves.

### Clinical Protocols

Clinical information was obtained from structured interviews and medical records. Routine laboratory measurements and screening for metabolic causes of intellectual impairment from blood and urine were obtained from a certified clinical diagnostic laboratory at Heidelberg University.

### Patient

The patient is the single child of healthy parents originating from Western Afghanistan that are consanguineous as 1^st^ degree cousins. Family history was reported as unremarkable despite further consanguineous marriages. Oligohydramnios was noted during the last trimenon of pregnancy, but birth occurred spontaneously, at term and with normal parameters. Walking was achieved by 22 months. At 3½ years expressive speech delay (ten active words), clumsiness, atactic gait and generalized mild muscular hypotonia were noted. There were no dysmorphic signs except for a hypopigmented skin area of 20×20 mm at the thoracal wall. Cranial MRI was unremarkable apart from an axial arachnoidal cyst of 29×18 mm in cisterna quadrigemina. Follow-up visits at 5½ and 6¼ years confirmed persistence of developmental, speech (∼60 active words, 2-word sentences) and coordination deficits. Recurrent otitis media was noted, but hearing tests were in the normal range. SON-R 2½-7 non-verbal intelligence testing revealed an overall IQ of 51 (CI:48–65) consistent with moderate mental retardation. The parents characterized the patient as showing low social competence, extensive fear towards novel situations and a preference for repetitive behaviors. Blood parameters indicative of metabolic causes of intellectual disability were in the normal range.

### Cell Culture

Peripheral venous blood lymphocytes from the patient, parents and a healthy male control were obtained, EBV-immortalized lymphoblasts generated and skin fibroblasts cultures (from the patient) generated according to routine protocols. In brief, lymphocytes and lymphoblastoid cell lines were maintained in RPMI 1640 culture medium (Gibco) supplemented with 10% heat-inactivated fetal bovine serum (Invitrogen), L-glutamine (Gibco), penicillin/streptomycin mix (Gibco), and non-essential amino acid solution (Gibco), until just before the medium was exhausted with around 75% confluence. Cells were then placed in fresh media and arrested in metaphase with 0.05 µg/ml colcemid (Invitrogen) [for karyotyping] or 0.1 µg/ml demecolcine (Sigma) [for flow cytometry] for 6 hours or overnight before harvesting.

### Cytogenetic and CNV Analyses

Chromosomes were obtained according to routine procedures and based on previously published protocols. [32]–[33] FISH analyses for fine mapping of chromosomal breakpoints were performed on 5–10 mitotic cells/marker using the following markers: SE7, CUTL1, D7S1503/D7S688/D7S1541, BAC3K23, pcp7q, YAC761H5, wcp2, wcp7, PAC892G20, RP11_542B5, RP11_16D24, RP1188K4, PR11_371N6. Genome-wide CNV analyses in a routine setting were performed using the Human Mapping 6.0 SNP-array (Affymetrix) according to established protocols.

### Chromosome Sorting and Flow Cytometry Analysis

Metaphase-blocked suspensions of cultured lymphoblasts were centrifuged at 1500 rpm at room temperature for 5 min. Cells were swollen by incubation for 10 min in 5 ml of hypotonic solution [75 mM KCl (Sigma), 0.5 mM spermidine (Sigma), 0.2 mM spermine (Sigma), 10 mM MgSO4.7H20, pH8.0]. Cell suspensions were centrifuged for 5 min at 1500 rpm. The cell pellet was carefully re-suspended in 3 ml of ice-cold polyamine isolation buffer [800 mM KCl, 5 mM EGTA (Sigma), 20 mM EDTA (Sigma), 150 mM Tris (Sigma), pH7.5]. After 15 min incubation on ice, the suspensions were vigorously vortexed for 10 s. Quality of chromosomes was evaluated by fluorescence microscopy after staining a small sample aliquot with DAPI (Invitrogen). Chromosome suspensions were briefly centrifuged for 3 min at 1200 rpm. Supernatants were collected for overnight staining at 4°C with 5 µg/ml Hoechst 33358 (Sigma) and 50 µg/ml chromomycin A3 (Sigma) in the presence of 10 mM MgSO4.7H2O, 10 mM sodium citrate and 25 mM sodium sulphite. Staining preparations were then filtered through a 20-µm filter (Celltrics, Partec) prior to analysis and sorting.

Stained chromosomes were analysed and sorted on a modified Moflo High Speed Sorter (Beckman Coulter) equipped with Coherent Sabre Argon and Krypton lasers. The Krypton laser configured to multiline UV (1W) was placed at the first laser tower and used as the MoFlo’s trigger beam. The Sabre Argon laser was configured to 457 nm (1W) and placed in the second laser position. A 351/20 nm bandpass filter (Semrock) was placed in front of the Moflo’s diode FSC detector. The Moflo’s optical bench was reconfigured with a −15PMT (Beckman Coulter) in the side scatter detector position of the L-configuration to collect the Hoechst fluorescence. A large width band pass was constructed in front of this detector by sandwiching a 364 nm RazonEdge longpass filter (Semrock) with a 439/154 nm BrightLine bandpass filter (Semrock). For the second laser position a 488 nm EdgeBasic long wave pass filter (Semrock) was placed in front of a −15PMT for the Chromomycin A3 fluorescence collection. The Moflo’s fluidics were fitted with a 70 µm nozzle using FACSFlow (BD Biosciences) as sheath fluid. The instrument was aligned using Flow-Check Fluorospheres (Beckman Coulter) and then fine aligned with the chromosomes. Offline analysis was performed using FlowJo (Treestar Inc.). Chromosomes were sorted into 1.5 ml low DNA binding Eppendorf tubes and stored at −20°C.

### Mate-pair and Paired-end Library Preparation and Next-generation Sequencing

DNA and RNA were extracted from blood lymphocytes according to routine protocols. DNA libraries for mate-pair sequencing were prepared according to Illumina protocol 1005363 Rev.B. using the Illumina 5 kb mate-pair sample prep kits (Illumina Cat# PE-112-2002). In short, 10 µg of genomic DNA was sheared into 5 kb fragment length using a Hydroshear (GeneMachines). Fragments were end repaired, biotin labelled and then size selected by gel electrophoresis to 5 kb after which they were circularized overnight. Linear DNA was removed by enzymatic digestion. The circularized DNA was then fragmented to produce ligated mate pair fragments which were isolated by Streptavidin-purification of the biotinylated DNA. Isolated fragments were end repaired, A-tailed and library adaptors were ligated. The adapter modified fragments were then enriched by PCR amplification (18 cycles). After amplification a further size selection step (to 500 bp) was performed to extract the correctly modified fragments. Mate-pair libraries of the patient were sequenced on an Illumina GAIIx for 2×36 cycles.

For high-resolution analysis of chromosomes 2 and der(2) of the patient, 1 µg of each of the two FACS-enriched DNA fractions (isolated according to above protocol) were prepared for paired-end library sequencing using Illumina paired-end library preparation kits according to the manufacturer’s instructions (Illumina). Sufficient quality of all libraries was ensured using an Agilent Bioanalyser 2100 (Agilent Technologies, Boeblingen, Germany). Chromosome 2 and der(2) enriched samples were sequenced on a HiSeq2000 for 1×103 read cycles (read 1) and 79 cycles (read 2).

### Data Analysis and Confirmation

All resulting sequence data were aligned to the hg19 build of the human reference genome using the ELAND aligner algorithm (Illumina). Mate-pair reads were analysed using custom-generated Perl scripts. In order to identify potential breakpoints of inter-chromosomal translocations, sequence read pairs were filtered for read pairs where individual reads aligned to different chromosomes. Intra-chromosomal inversions and deletions were identified by querying the mate-pair read data for read pairs aligning with cut-offs at 10 kb (2× library insert size). Read pairs which fell into the above categories were clustered together. Clusters containing at least six overlapping read pairs where considered as potentially real and manually curated. Curated clusters were mapped to gene coordinates of the Ensembl human reference genome build 72 (www.ensembl.org). Regions overlapping with HGNC-annotated genes where structural variation was expected to impact on respective gene products were PCR-amplified and PCR amplicons were Sanger sequenced (GATC, Konstanz, Germany). Presence of abnormal gene products was validated by amplifying proposed fusion mRNAs isolated from patient and control lymphoblasts by quantitative-reverse polymerase chain reaction (qRT-PCR) using SYBR Green Supermix (Bio-Rad, Hercules, CA) according to established protocols. Primer sequences are available on request. Position of potentially clinically relevant SVs and phenotype information on the index patient have been submitted to the European Bioinformatics Institute’s Database of Genomic Variants archive (http://www.ebi.ac.uk/dgva), accession number estd210. Further data can be made available to researchers on request to the authors.

## Supporting Information

Figure S1 (EPS)

Figure S2 (EPS)

Table S1 (PDF)

Table S2 (PDF)
