# Supplementary material for: Sequencing of a Patient with Balanced Chromosome Abnormalities and Neurodevelopmental Disease Identifies Disruption of Multiple High Risk Loci by Structural Variation
Source: PLoS One. 2014 Mar 13;9(3):e90894. doi: 10.1371/journal.pone.0090894 (PMC3953210; doi:10.1371/journal.pone.0090894)
Supplement: Table S1 — (PDF) [file pone.0090894.s003.pdf]

Table S1

Mate-pair sequencing-proposed structural variants of >10kb in size mapped to near or within annotated genes across the genome of the index patient (Ensembl 72)

chromosome	variant no.	5' boundary	3' boundary	read depth	size [bp]	SV	Ensembl ID	gene start	gene end	GeneName	type
chr1	1	2583845	2689356	38	105511	DEL	ENSG00000215912	2567415	2718286	**TTC34**	intronic
chr1	2	72762870	72816455	12	53585	DEL	ENSG00000227207	72767155	72767512	**ENSG00000227207**	pseudogene
chr1	3	102351140	102368152	9	17012	DEL	ENSG00000162699	102358661	102359603	**DNAJA1P5**	pseudogene
chr1	3	102351140	102368152	9	17012	DEL	ENSG00000118733	102268130	102462586	**OLFM3**	intronic
chr1	4	144891176	144908295	6	17119	DEL	ENSG00000178104	144836157	145076186	**PDE4DIP**	intronic
chr1	5	174792396	174805858	9	13462	DEL	ENSG00000152061	174128548	174964445	**RABGAP1L**	intronic
chr1	6	184810129	184825656	10	15527	DEL	ENSG00000135842	184759858	184943682	**FAM129A**	intronic
chr1	7	229807882	229825033	13	17151	DEL	ENSG00000213028	229824746	229825651	**ENSG00000213028**	pseudogene
chr1	8	247847031	247861292	11	14261	DEL	ENSG00000235749	247803051	247910364	**ENSG00000235749**	intronic
chr1	9	248048260	248062520	14	14260	DEL	ENSG00000238243	248031277	248060449	**OR2W3**	intronic
chr10	10	47018242	47064611	140	46369	INV	ENSG00000150165	47011753	47174093	**ANXA8L1**	intronic
chr10	11	111567051	111582236	17	15185	DEL	ENSG00000224907	111567409	111567887	**ENSG00000224907**	pseudogene
chr11	12	1910784	1964943	92	54159	DEL	ENSG00000184682	1910375	1912084	**C11orf89**	unclear
chr11	12	1910784	1964943	92	54159	DEL	ENSG00000229671	1923877	1929145	**ENSG00000229671**	linc/transcr.
chr11	12	1910784	1964943	92	54159	DEL	ENSG00000252187	1941339	1941488	**ENSG00000252187**	unclear
chr11	12	1910784	1964943	92	54159	DEL	ENSG00000130592	1874200	1913497	**LSP1**	unclear
chr11	12	1910784	1964943	92	54159	DEL	ENSG00000130595	1940792	1959936	**TNNT3**	unclear
chr11	13	6088619	6101135	13	12516	DEL	ENSG00000234895	6088103	6089016	**OR52X1P**	pseudogene
chr11	14	29003837	29017896	19	14059	DEL	ENSG00000249867	28724162	29085368	**ENSG00000249867**	intronic
chr11	15	69088711	69141936	16	53225	DEL	ENSG00000172927	69061605	69182494	**MYEOV**	intronic
chr11	16	92865645	92879666	14	14021	DEL	ENSG00000180773	92877341	92931130	**SLC36A4**	intergenic
chr11	17	101562129	101579339	17	17210	DEL	ENSG00000137672	101322295	101743293	**TRPC6**	intronic
chr11	18	114420698	114432410	7	11712	DEL	ENSG00000095110	114392437	114430617	**NXPE1**	intronic
chr12	19	11190412	11254341	12	63929	DEL	ENSG00000256400	11249778	11249873	**ENSG00000256400**	unclear
chr12	19	11190412	11254341	12	63929	DEL	ENSG00000111215	10977559	11324212	**PRR4**	unclear
chr12	19	11190412	11254341	12	63929	DEL	ENSG00000212127	11090005	11324172	**TAS2R14**	unclear
chr12	19	11190412	11254341	12	63929	DEL	ENSG00000255374	11243886	11244912	**TAS2R43**	unclear
chr12	19	11190412	11254341	12	63929	DEL	ENSG00000226761	11213964	11214893	**TAS2R46**	unclear
chr12	19	11190412	11254341	12	63929	DEL	ENSG00000256019	11200931	11201855	**TAS2R63P**	unclear
chr12	19	11190412	11254341	12	63929	DEL	ENSG00000256274	11229368	11231770	**TAS2R64P**	unclear
chr12	20	43016464	43030165	8	13701	DEL	ENSG00000257510	43009305	43040300	**ENSG00000257510**	linc/transcr.
chr12	20	43016464	43030165	8	13701	DEL	ENSG00000257687	43020810	43020939	**ENSG00000257687**	pseudogene
chr12	21	45899907	45914382	12	14475	DEL	ENSG00000257657	45912101	46004403	**ENSG00000257657**	intronic
chr12	22	99792360	99806826	8	14466	DEL	ENSG00000185046	99120235	100378432	**ANKS1B**	intronic
chr13	23	51064709	51076629	10	11920	DEL	ENSG00000176124	50656307	51297372	**DLEU1**	intronic
chr13	24	114323378	114427858	21	104480	DEL	ENSG00000185974	114321594	114438637	**GRK1**	intronic
chr15	25	29157401	29210439	13	53038	DEL	ENSG00000034053	29129629	29410518	**APBA2**	intronic
chr15	26	83547144	83562742	20	15598	DEL	ENSG00000103942	83509838	83654661	**HOMER2**	intronic
chr16	27	18828787	18843173	10	14386	DEL	ENSG00000157106	18816175	18937776	**SMG1**	intronic
chr16	28	75235803	75261230	35	25427	INV	ENSG00000240338	75259972	75262093	**ENSG00000240338**	linc/transcr.
chr16	29	83666054	83681468	15	15414	DEL	ENSG00000140945	82660408	83830204	**CDH13**	intronic
chr17	30	21208079	21225633	20	17554	DEL	ENSG00000034152	21187984	21218552	**MAP2K3**	intronic
chr17	31	39199154	39216570	9	17416	DEL	ENSG00000212725	39202793	39203568	**KRTAP2-1**	intergenic
chr17	31	39199154	39216570	9	17416	DEL	ENSG00000214518	39210750	39211482	**KRTAP2-2**	intergenic
chr17	31	39199154	39216570	9	17416	DEL	ENSG00000212724	39215495	39216344	**KRTAP2-3**	intergenic
chr17	32	41382048	41400874	35	18826	DEL	ENSG00000236383	41368960	41383338	**ENSG00000236383**	intergenic
chr17	33	41400914	41466407	19	65493	INV	ENSG00000188825	41447213	41466567	**ENSG00000188825**	linc/transcr.
chr19	34	19829848	19843750	9	13902	DEL	ENSG00000105708	19821280	19843906	**ZNF14**	intronic
chr19	35	35847100	35867440	15	20340	DEL	ENSG00000185897	35849362	35851387	**FFAR3**	intergenic
chr19	35	35847100	35867440	15	20340	DEL	ENSG00000126251	35861831	35863855	**GPR42**	intergenic
chr19	36	39259961	39284385	11	24424	INV	ENSG00000182472	39220827	39260544	**CAPN12**	intronic
chr19	37	52129340	52154291	28	24951	DEL	ENSG00000243469	52152115	52152863	**RPL7P51**	pseudogene
chr19	37	52129340	52154291	28	24951	DEL	ENSG00000254415	52145806	52150054	**SIGLEC14**	unclear
chr19	37	52129340	52154291	28	24951	DEL	ENSG00000105501	52114781	52150151	**SIGLEC5**	unclear
chr19	38	54796983	54810383	9	13400	DEL	ENSG00000240197	54797902	54798219	**ENSG00000240197**	pseudogene
chr19	38	54796983	54810383	9	13400	DEL	ENSG00000251431	54808714	54809423	**ENSG00000251431**	pseudogene
chr19	38	54796983	54810383	9	13400	DEL	ENSG00000170866	54799854	54809952	**LILRA3**	coding hit
chr2	39	22346680	162195830	21	139849150	INV	ENSG00000231200	22156208	22753977	**ENSG00000231200**	coding hit
chr2	39	22346680	162195830	21	139849150	INV	ENSG00000115233	162164549	162268228	**PSMD14**	coding hit
chr2	40	48847148	48862880	11	15732	DEL	ENSG00000239995	48859995	48860492	**ENSG00000239995**	pseudogene
chr2	40	48847148	48862880	11	15732	DEL	ENSG00000242441	48844937	48960287	**GTF2A1L**	intronic
chr2	40	48847148	48862880	11	15732	DEL	ENSG00000138039	48859428	48982880	**LHCGR**	intronic
chr2	40	48847148	48862880	11	15732	DEL	ENSG00000068781	48757064	49003654	**STON1-GTF2A1L**	intronic
chr2	41	110105277	110255519	30	150242	DEL	ENSG00000172985	109745804	110262207	**SH3RF3**	intronic
chr2	42	149686463	149794229	24	107766	DEL	ENSG00000168280	149632819	149883273	**KIF5C**	intronic
chr2	43	185675948	188166401	24	2490453	INV	ENSG00000224063	187867947	188419390	**ENSG00000224063**	linc/transcr.
chr2	43	185675948	188166401	24	2490453	INV	ENSG00000170396	185463093	185804219	**ZNF804A**	coding hit
chr2	44	242849529	243040821	13	191292	DEL	ENSG00000220804	243030784	243102304	**ENSG00000220804**	linc/transcr.
chr2	44	242849529	243040821	13	191292	DEL	ENSG00000226423	242967334	242968327	**ENSG00000226423**	linc/transcr.
chr2	44	242849529	243040821	13	191292	DEL	ENSG00000232002	242989846	243026289	**ENSG00000232002**	linc/transcr.
chr2	44	242849529	243040821	13	191292	DEL	ENSG00000233806	242823514	243020873	**ENSG00000233806**	linc/transcr.
chr2	44	242849529	243040821	13	191292	DEL	ENSG00000237940	242912834	242919427	**ENSG00000237940**	linc/transcr.
chr2	44	242849529	243040821	13	191292	DEL	ENSG00000261186	243029505	243030608	**ENSG00000261186**	linc/transcr.
chr21	45	42952285	43009493	14	57208	DEL	ENSG00000223400	42948062	42953246	**ENSG00000223400**	intergenic
chr22	46	24270522	24314151	17	43629	DEL	ENSG00000206090	24282185	24284985	**ENSG00000206090**	pseudogene
chr22	46	24270522	24314151	17	43629	DEL	ENSG00000225282	24269087	24271761	**ENSG00000225282**	pseudogene
chr22	46	24270522	24314151	17	43629	DEL	ENSG00000228039	24305942	24306644	**ENSG00000228039**	pseudogene
chr22	46	24270522	24314151	17	43629	DEL	ENSG00000231271	24292105	24296229	**ENSG00000231271**	pseudogene
chr22	46	24270522	24314151	17	43629	DEL	ENSG00000099977	24313554	24322660	**DDT**	intergenic
chr22	46	24270522	24314151	17	43629	DEL	ENSG00000099974	24309089	24314721	**DDTL**	intergenic
chr22	46	24270522	24314151	17	43629	DEL	ENSG00000133433	24299601	24303373	**GSTT2B**	intronic
chr3	47	8215221	8228706	10	13485	DEL	ENSG00000227110	7994492	8653610	**LMCD1-AS1**	intronic
chr3	48	20308321	20322485	10	14164	DEL	ENSG00000199577	20310457	20310562	**ENSG00000199577**	intergenic
chr3	49	22089382	22101893	13	12511	DEL	ENSG00000151789	21459915	22414812	**ZNF385D**	intronic
chr3	50	37975145	37990792	11	15647	DEL	ENSG00000144677	37903451	38025960	**CTDSPL**	intronic
chr4	51	21156407	21172438	14	16031	DEL	ENSG00000185774	20730239	21950422	**KCNIP4**	intronic
chr4	52	80885055	80899003	15	13948	DEL	ENSG00000163297	80822303	81046608	**ANTXR2**	intronic
chr4	53	91592561	91607363	19	14802	DEL	ENSG00000251401	91604147	91604274	**ENSG00000251401**	pseudogene
chr4	53	91592561	91607363	19	14802	DEL	ENSG00000184305	91048686	92523064	**CCSER1**	intronic
chr4	54	98169851	98191877	11	22026	DEL	ENSG00000163116	98105244	99064391	**STPG2**	intronic
chr4	55	167673360	167685308	12	11948	DEL	ENSG00000196104	167654535	168155947	**SPOCK3**	intronic
chr4	56	173421510	173438260	10	16750	DEL	ENSG00000174473	172733405	173962710	**GALNTL6**	intronic
chr4	57	190581342	190617844	70	36502	INV	ENSG00000250739	190580759	190582640	**ENSG00000250739**	linc/transcr.
chr5	58	21524396	29427910	10	7903514	INV	ENSG00000183666	21341942	21544886	**GUSBP1**	pseudogene
chr5	59	97047347	97099642	6	52295	DEL	ENSG00000251193	97073598	97074617	**ENSG00000251193**	pseudogene
chr5	60	103849914	103865441	15	15527	DEL	ENSG00000251574	103718999	104728671	**ENSG00000251574**	intronic
chr5	61	151451978	151466567	21	14589	DEL	ENSG00000254226	151329132	151650009	**ENSG00000254226**	intronic
chr6	62	24807140	24820110	15	12970	DEL	ENSG00000111913	24797601	25042238	**FAM65B**	intronic
chr6	63	51735042	51750452	15	15410	DEL	ENSG00000170927	51480098	51952423	**PKHD1**	intronic
chr6	64	53925807	53940056	25	14249	DEL	ENSG00000146147	53794780	54131078	**MLIP**	intronic
chr6	64	53925807	53940056	25	14249	DEL	ENSG00000235050	53893975	53944524	**MLIP-AS1**	intronic
chr6	65	57418880	57433932	23	15052	DEL	ENSG00000146143	57179603	57513375	**PRIM2**	intronic
chr7	66	147371	165206	19	17835	DEL	ENSG00000240859	149597	155465	**ENSG00000240859**	linc/transcr.
chr7	66	147371	165206	19	17835	DEL	ENSG00000242474	135853	149466	**ENSG00000242474**	intergenic
chr7	66	147371	165206	19	17835	DEL	ENSG00000261795	161765	164972	**ENSG00000261795**	intergenic
chr7	67	91027715	91046421	10	18706	DEL	ENSG00000235450	91010093	91186163	**ENSG00000235450**	intronic
chr7	67	91027715	91046421	10	18706	DEL	ENSG00000243144	90940683	91144724	**ENSG00000243144**	intronic
chr7	68	93413402	93427455	7	14053	DEL	ENSG00000127928	93220885	93540577	**ENSG00000127928**	intronic
chr7	69	100548501	100612424	43	63923	DEL	ENSG00000225946	100606878	100611410	**ENSG00000225946**	intergenic
chr7	69	100548501	100612424	43	63923	DEL	ENSG00000228273	100547257	100550424	**ENSG00000228273**	intergenic
chr7	69	100548501	100612424	43	63923	DEL	ENSG00000169894	100547187	100611118	**MUC3A**	intronic
chr7	70	154390010	154401624	8	11614	DEL	ENSG00000130226	153584182	154685995	**DPP6**	intronic
chr8	71	591156	604507	22	13351	DEL	ENSG00000104714	564746	688106	**ERICH1**	intronic
chr8	72	58116724	58127885	6	11161	DEL	ENSG00000253301	58055248	58152857	**ENSG00000253301**	intronic
chr8	73	73783275	73799310	35	16035	DEL	ENSG00000253784	73787094	73793975	**ENSG00000253784**	intergenic
chr8	73	73783275	73799310	35	16035	DEL	ENSG00000182674	73449626	73850584	**KCNB2**	intronic
chr8	74	129461762	129476220	19	14458	DEL	ENSG00000254275	129417515	129576925	**ENSG00000254275**	intronic
chr8	75	144696615	144719270	13	22655	DEL	ENSG00000254812	144714241	144715926	**ENSG00000254812**	linc/transcr.
chr8	75	144696615	144719270	13	22655	DEL	ENSG00000104522	144694788	144700218	**TSTA3**	intergenic
chr8	75	144696615	144719270	13	22655	DEL	ENSG00000183309	144718183	144735900	**ZNF623**	intergenic
chr9	76	15812105	15826481	22	14376	DEL	ENSG00000164989	15552895	16061661	**CCDC171**	intronic
chr9	77	71733856	71747590	24	13734	DEL	ENSG00000227410	71736209	71737148	**ENSG00000227410**	intergenic
chr9	77	71733856	71747590	24	13734	DEL	ENSG00000119139	71736224	71870124	**TJP2**	intronic
chrX	78	46807299	46833483	21	26184	INV	ENSG00000102221	46771711	46920641	**PHF16**	intronic
chrX	79	149568049	149584165	8	16116	INV	ENSG00000013619	149529689	149682448	**MAMLD1**	intronic
chrY	80	13459801	13490013	143	30212	DEL	ENSG00000258567	13462594	13463857	**DUX4L16**	pseudogene
chrY	80	13459801	13490013	143	30212	DEL	ENSG00000259154	13470597	13471863	**DUX4L17**	pseudogene
chrY	80	13459801	13490013	143	30212	DEL	ENSG00000259029	13477233	13478499	**DUX4L18**	pseudogene
chrY	80	13459801	13490013	143	30212	DEL	ENSG00000258991	13488005	13489271	**DUX4L19**	pseudogene
