# Supplementary material for: Sequencing of a Patient with Balanced Chromosome Abnormalities and Neurodevelopmental Disease Identifies Disruption of Multiple High Risk Loci by Structural Variation
Source: PLoS One. 2014 Mar 13;9(3):e90894. doi: 10.1371/journal.pone.0090894 (PMC3953210; doi:10.1371/journal.pone.0090894)
Supplement: Table S2 — (PDF) [file pone.0090894.s004.pdf]

Table S2

Homozygous coding missense variants identified by deep sequencing of chromosomes 2 and der(2)

* allele frequencies

variant no.	GeneName	rsid	chr	start	end	REF	ALT	geneotype	qual	depth	1000G*	NHLBI6500*	GERP++	EXONIC info	Impact	SIFT	PP2
1	**TAF1B**	rs396190	chr2	10045024	10045024	G	A	hom	43	43	0.95	0.930801	3.27	nonsynonymous	TAF1B:NM_005680:e	1	0
2	**RAD51AP2**	rs834514	chr2	17696573	17696573	C	T	hom	64.5	43	0.58	0.511555	2.29	nonsynonymous	RAD51AP2:NM_001(	0.38	0.979
3	**GEN1**	rs1812152	chr2	17942775	17942775	T	A	hom	179	47	0.79	0.671229	n	nonsynonymous	GEN1:NM_00113000	0.53	0
4	**GEN1**	rs300175	chr2	17954027	17954027	G	A	hom	32	45	0.99	0.983546	n	nonsynonymous	GEN1:NM_00113000	0.47	0
5	**GEN1**	rs300169	chr2	17962518	17962518	C	T	hom	153	44	0.74	0.618714	n	nonsynonymous	GEN1:NM_00113000	0.92	0
6	**MATN3**	rs28939676	chr2	20202930	20202930	G	A	hom	205	29	0.01	0.011459	5.5	nonsynonymous	MATN3:NM_002381:(	0	n
7	**APOB**	rs584542	chr2	21232803	21232803	T	C	hom	209	43	0.97	0.981624	n	nonsynonymous	APOB:NM_000384:e:	0.77	0.009
8	**APOB**	rs568413	chr2	21235475	21235475	T	C	hom	157	38	1	0.999692	n	nonsynonymous	APOB:NM_000384:e:	0.65	0
9	**GPR113**	rs7568529	chr2	26534801	26534801	G	A	hom	3.41	22	0.72	0.684915	5.18	nonsynonymous	GPR113:NM_001145	0.04	0.995
10	**GPR113**	rs2052937	chr2	26536694	26536694	C	T	hom	194	26	0.71	0.689835	3.47	nonsynonymous	GPR113:NM_001145	0.42	0
11	**ABHD1**	rs6715286	chr2	27351948	27351948	T	G	hom	118	26	0.99	0.983469	n	nonsynonymous	ABHD1:NM_032604:(	1	0
12	**FAM179A**	rs11127202	chr2	29240060	29240060	A	G	hom	23	25	0.21	0.233414	3.85	nonsynonymous	FAM179A:NM_19928	1	n
13	**ALK**	rs1881420	chr2	29416481	29416481	T	C	hom	5.29	21	0.4	0.223358	n	nonsynonymous	ALK:NM_004304:exo	0.31	0.123
14	**ALK**	rs1670283	chr2	29416572	29416572	T	C	hom	21.8	29	0.99	0.990312	n	nonsynonymous	ALK:NM_004304:exo	1	0
15	**BIRC6**	rs60197615	chr2	32667182	32667182	G	C	hom	163	43	1	n	5.64	nonsynonymous	BIRC6:NM_016252:e	1	n
16	**BIRC6**	rs2366894	chr2	32713706	32713706	A	T	hom	95	41	0.58	0.658698	4.48	nonsynonymous	BIRC6:NM_016252:e	0.97	n
17	**LTBP1**	rs4422143	chr2	33585796	33585796	T	C	hom	136	37	1	0.999923	4.89	nonsynonymous	LTBP1:NM_0011662(	1	0
18	**SULT6B1**	rs10205833	chr2	37406680	37406680	C	G	hom	54.3	37	0.41	0.249731	n	nonsynonymous	SULT6B1:NM_00103:	1	0
19	**CEBPZ**	rs2098386	chr2	37456032	37456032	C	T	hom	39	40	0.93	0.879499	n	nonsynonymous	CEBPZ:NM_005760:(	1	0.127063
20	**RMDN2**	rs4670800	chr2	38179134	38179134	G	A	hom	59.5	51	1	n	n	nonsynonymous	RMDN2:NM_144713:	0.77	0
21	**CYP1B1**	rs1800440	chr2	38298139	38298139	T	C	hom	36	39	0.1	0.134938	5.95	nonsynonymous	CYP1B1:NM_000104	0.01	0.903
22	**CYP1B1**	rs1056836	chr2	38298203	38298203	C	G	hom	157	39	0.61	0.445564	5.95	nonsynonymous	CYP1B1:NM_000104	0.34	0
23	**DHX57**	rs7598922	chr2	39082344	39082344	T	C	hom	87.1	49	0.6	0.535907	n	nonsynonymous	DHX57:NM_198963:(	0.49	0
24	**EML4**	rs10202624	chr2	42515388	42515388	A	G	hom	140	46	0.85	0.917333	5.67	nonsynonymous	EML4:NM_00114507(	0.85	0
25	**HAAO**	rs3816183	chr2	43015719	43015719	T	C	hom	222	22	0.82	0.793634	3.66	nonsynonymous	HAAO:NM_012205:e:	1	0.067046
26	**ABCG8**	rs6544718	chr2	44104925	44104925	T	C	hom	58.3	35	0.89	0.837229	n	nonsynonymous	ABCG8:NM_022437:(	1	0
27	**FSHR**	rs6165	chr2	49191041	49191041	C	T	hom	20	42	0.51	0.454636	n	nonsynonymous	FSHR:NM_181446:e:	0.42	0.07985
28	**PSME4**	rs805408	chr2	54120025	54120025	A	T	hom	38	45	0.3	0.271106	5.41	nonsynonymous	PSME4:NM_014614:(	0.75	0.709923
29	**C2orf73**	rs2280718	chr2	54587596	54587596	C	T	hom	4.61	39	0.73	0.761328	3.18	nonsynonymous	C2orf73:NM_001100:	0.51	n
30	**CLHC1**	rs14026	chr2	55404794	55404794	G	A	hom	218	51	0.5	0.511304	n	nonsynonymous	CLHC1:NM_0011355	1	0
31	**CLHC1**	rs6716066	chr2	55407754	55407754	C	T	hom	172	44	0.97	0.982854	3.59	nonsynonymous	CLHC1:NM_0011355	1	0.438892
32	**MTIF2**	rs11357	chr2	55470115	55470115	C	T	hom	153	35	0.68	0.690278	2.14	nonsynonymous	MTIF2:NM_0010053(	1	0
33	**SMEK2**	rs2903704	chr2	55805440	55805440	T	C	hom	189	33	0.99	n	n	nonsynonymous	SMEK2:NM_0011229	1	n
34	**PNPT1**	rs782572	chr2	55912120	55912120	T	C	hom	21.8	29	0.38	0.446871	n	nonsynonymous	PNPT1:NM_033109:(	0.19	0
35	**KIAA1841**	n	chr2	61361327	61361327	G	-	hom	214	49	n	n	n	frameshift deletion	KIAA1841:NM_0325(	n	n
36	**C2orf74**	rs1729674	chr2	61389737	61389737	T	G	hom	151	43	0.43	n	2.31	nonsynonymous	C2orf74:NM_001143(	0	n
37	**USP34**	rs6722430	chr2	61575308	61575308	A	G	hom	179	35	0.99	0.985651	3.83	nonsynonymous	USP34:NM_014709:(	1	0
38	**ETAA1**	rs3770657	chr2	67630980	67630980	G	A	hom	49	45	0.72	0.761538	3.4	nonsynonymous	ETAA1:NM_019002:(	1	0
39	**ETAA1**	rs3770655	chr2	67632125	67632125	C	T	hom	54.3	47	0.73	0.775958	n	nonsynonymous	ETAA1:NM_019002:(	0.82	0
40	**FIGLA**	rs7566476	chr2	71012734	71012734	C	G	hom	178	28	0.75	0.626685	3.24	nonsynonymous	FIGLA:NM_00100431	1	n
41	**CD207**	rs741326	chr2	71058835	71058835	A	G	hom	163	27	0.54	0.437219	n	nonsynonymous	CD207:NM_015717:(	0.75	n
42	**ALMS1**	rs2037814	chr2	73675669	73675669	T	G	hom	50	32	0.86	0.880566	n	nonsynonymous	ALMS1:NM_015120:(	0.14	0
43	**ALMS1**	n	chr2	73675844	73675844	C	G	hom	17.8	35	n	n	n	nonsynonymous	ALMS1:NM_015120:(	1	0.001
44	**NAT8B**	rs4852974	chr2	73927931	73927931	A	G	hom	26.8	32	0.74	0.74927	3.18	unknown	UNKNOWN	n	n
45	**NAT8B**	rs2001490	chr2	73928098	73928098	C	G	hom	31	29	0.58	0.561126	2.4	unknown	UNKNOWN	n	n
46	**POLR1A**	rs4832242	chr2	86317037	86317037	G	C	hom	37	44	0.76	0.787864	n	nonsynonymous	POLR1A:NM_015425	0.62	0.001
47	**IMMT**	rs1050301	chr2	86400824	86400824	G	A	hom	39.3	41	0.33	0.260836	3.43	nonsynonymous	IMMT:NM_00110016(	0.04	n
48	**KDM3A**	rs2030259	chr2	86683642	86683642	A	G	hom	138	27	0.85	0.801784	3.51	nonsynonymous	KDM3A:NM_0011466	0.95	0
49	**ASTL**	rs749458	chr2	96795857	96795857	T	C	hom	64.3	24	0.89	0.850531	n	nonsynonymous	ASTL:NM_00100203(	1	0
50	**ANKRD36**	rs200480616	chr2	97823853	97823853	G	C	hom	98	22	n	0.01972	n	nonsynonymous	ANKRD36:NM_0011(	n	n
51	**AFF3**	rs4851223	chr2	100343557	100343557	C	T	hom	44	53	1	n	5.26	nonsynonymous	AFF3:NM_00102510(	1	0
52	**RFX8**	rs4851436	chr2	102038849	102038849	T	A	hom	218	46	0.89	0.947657	4.74	nonsynonymous	RFX8:NM_00114566(	0.29	n
53	**TMEM182**	rs887987	chr2	103431404	103431404	T	C	hom	222	38	0.99	0.975012	2.93	nonsynonymous	TMEM182:NM_1446:	1	0
54	**GCC2**	rs2718698	chr2	109099572	109099572	C	G	hom	19	51	1	0.990005	5.13	nonsynonymous	GCC2:NM_181453:e:	1	0
55	**CKAP2L**	rs6731822	chr2	113513825	113513825	T	C	hom	210	34	0.97	0.940181	n	nonsynonymous	CKAP2L:NM_152515	0.66	0.071
56	**IL36A**	rs895497	chr2	113763575	113763575	A	G	hom	43.5	43	0.82	0.725041	n	nonsynonymous	IL36A:NM_014440:e:	0.36	0
57	**DPP10**	rs1446495	chr2	116525960	116525960	G	A	hom	127	31	0.97	0.969245	5.25	nonsynonymous	DPP10:NM_020868:(	1	0
58	**C2orf76**	rs1052500	chr2	120060082	120060082	T	C	hom	40.5	55	0.66	0.724126	n	nonsynonymous	C2orf76:NM_001017(	0.6	0
59	**PCDP1**	rs2272058	chr2	120388412	120388412	G	A	hom	55.3	47	0.98	0.977241	n	nonsynonymous	PCDP1:NM_0012710	0.77	0.002
60	**TMEM177**	rs1983406	chr2	120439230	120439230	C	G	hom	27	22	0.78	0.771106	n	nonsynonymous	TMEM177:NM_0011(	0.81	0
61	**TUBA3E**	rs10208844	chr2	130949411	130949411	G	T	hom	76.3	24	0.9	0.947769	2.64	nonsynonymous	TUBA3E:NM_207312	1	0
62	**POTEI**	rs4369872	chr2	131221170	131221170	C	T	hom	29.8	22	n	n	n	nonsynonymous	POTEI:NM_0012774(	0	n
63	**R3HDM1**	rs961360	chr2	136393658	136393658	A	G	hom	22.8	58	0.31	0.183377	5.58	nonsynonymous	R3HDM1:NM_01536(	0.36	0.578
64	**LCT**	rs4954449	chr2	136575534	136575534	T	C	hom	25.8	23	0.98	0.978395	5.56	nonsynonymous	LCT:NM_002299:exo	1	0
65	**LCT**	rs3754689	chr2	136590746	136590746	C	T	hom	160	29	0.34	0.27026	2.81	nonsynonymous	LCT:NM_002299:exo	1	0.024
66	**NEB**	rs6710212	chr2	152490458	152490458	A	G	hom	21	36	0.98	0.97569	6.02	nonsynonymous	NEB:NM_001164507:	n	n
67	**NEB**	rs6711382	chr2	152531077	152531077	A	G	hom	222	50	0.74	0.817781	5.52	nonsynonymous	NEB:NM_001164507:	0.18	n
68	**NEB**	rs6735208	chr2	152536498	152536498	T	A	hom	45.5	38	0.62	0.698568	n	nonsynonymous	NEB:NM_001164507:	0.22	n
69	**GPD2**	rs2116665	chr2	157406249	157406249	G	A	hom	137	33	0.66	0.630094	3.17	nonsynonymous	GPD2:NM_00108311	0.2	0.01
70	**CCDC148**	rs7559772	chr2	159166069	159166069	T	C	hom	18	40	0.95	0.947024	3.84	nonsynonymous	CCDC148:NM_1388(	1	0
71	**TANC1**	rs12466551	chr2	160019863	160019863	A	G	hom	45.3	37	0.98	0.987299	5.16	nonsynonymous	TANC1:NM_033394:(	1	0
72	**WDSUB1**	rs174264	chr2	160136337	160136337	G	A	hom	48.5	40	1	0.999923	5.79	nonsynonymous	WDSUB1:NM_15252	1	0
73	**LY75,LY75-CD302**	rs12692566	chr2	160676427	160676427	C	A	hom	27	36	0.86	0.848692	4.5	nonsynonymous	LY75:NM_002349:ex	1	0.252006
74	**TTC21B**	rs7592429	chr2	166788336	166788336	T	C	hom	211	58	0.95	0.945717	2.14	nonsynonymous	TTC21B:NM_024753	1	0.005122
75	**TTC21B**	rs1432273	chr2	166797646	166797646	C	T	hom	184	34	0.67	0.60203	2.52	nonsynonymous	TTC21B:NM_024753	0.01	0.651689
76	**SCN9A**	rs6746030	chr2	167099158	167099158	A	G	hom	206	30	0.89	0.877448	3.83	nonsynonymous	SCN9A:NM_002977:(	1	n
77	**SCN7A**	rs7565062	chr2	167334085	167334085	G	T	hom	31	51	0.69	0.701658	n	nonsynonymous	SCN7A:NM_002976:(	1	n
78	**NOSTRIN**	rs479661	chr2	169721377	169721377	G	A	hom	23	48	0.89	0.866756	4.96	nonsynonymous	NOSTRIN:NM_00103	1	0.420774
79	**LRP2**	rs2075252	chr2	170010985	170010985	T	C	hom	49	50	0.74	0.801322	5.55	nonsynonymous	LRP2:NM_004525:ex	1	0
80	**MYO3B**	rs6736609	chr2	171260787	171260787	G	A	hom	165	28	0.79	0.844545	n	nonsynonymous	MYO3B:NM_138995:	0.2	0.001
81	**METTL8**	rs10205459	chr2	172180771	172180771	A	G	hom	32	38	1	0.997591	5.63	unknown	UNKNOWN	0.62	n
82	**CYBRD1**	rs10455	chr2	172411273	172411273	G	A	hom	73.3	47	0.65	0.733892	n	nonsynonymous	CYBRD1:NM_00125(	0.56	0
83	**PDE11A**	rs6433711	chr2	178936614	178936614	C	T	hom	18.8	27	0.99	0.992619	5.28	nonsynonymous	PDE11A:NM_016953	1	0.002
84	**TTN**	rs12463674	chr2	179432185	179432185	A	G	hom	67.5	45	0.15	0.225855	5.65	nonsynonymous	TTN:NM_001256850:	n	n
85	**TTN**	rs4145333	chr2	179444768	179444768	C	G	hom	48	41	0.99	0.995617	n	nonsynonymous	TTN:NM_001256850:	n	n
86	**TTN**	rs36051007	chr2	179545859	179545859	C	T	hom	150	48	0.15	0.226359	n	nonsynonymous	TTN:NM_001256850:	n	n
87	**TTN**	rs2244492	chr2	179554305	179554305	C	T	hom	42	54	0.37	0.43349	5.34	nonsynonymous	TTN:NM_001256850:	0.69	n
88	**TTN**	rs922984	chr2	179615887	179615887	T	C	hom	19	47	0.78	0.818063	4.54	nonsynonymous	TTN:NM_133379:exo	0.03	0.563193
89	**TTN**	rs922985	chr2	179615931	179615931	C	G	hom	171	48	0.98	0.972453	3.9	nonsynonymous	TTN:NM_133379:exo	0.05	0.411494
90	**TTN**	rs7585334	chr2	179620951	179620951	C	T	hom	39.3	45	0.84	0.90281	4.2	nonsynonymous	TTN:NM_001267550:	n	n
91	**TTN**	rs6433728	chr2	179621477	179621477	C	T	hom	222	45	1	0.999169	n	nonsynonymous	TTN:NM_001267550:	n	n
92	**TTN**	rs2291310	chr2	179623758	179623758	C	T	hom	20.8	60	0.84	0.902353	n	nonsynonymous	TTN:NM_001256850:	0.73	0.209507
93	**TTN**	rs2291311	chr2	179629461	179629461	C	T	hom	222	37	0.84	0.896509	4.82	nonsynonymous	TTN:NM_001256850:	0.24	0.41084
94	**TTN**	rs1552280	chr2	179644035	179644035	G	A	hom	77.3	35	0.93	0.950861	5.72	nonsynonymous	TTN:NM_001256850:	0.35	0.350141
95	**TTN**	rs10497520	chr2	179644855	179644855	T	C	hom	32	54	0.55	0.734892	4.56	nonsynonymous	TTN:NM_001256850:	0.45	0.383601
96	**CWC22**	rs6433818	chr2	180810264	180810264	A	T	hom	174	46	0.94	0.896459	n	nonsynonymous	CWC22:NM_020943:	1	n
97	**NEUROD1**	rs1801262	chr2	182543455	182543455	T	C	hom	74.1	48	0.73	0.669383	4.43	nonsynonymous	NEUROD1:NM_0025	0.78	0.075581
98	**DNAJC10**	rs6729801	chr2	183584755	183584755	G	A	hom	36	40	0.36	0.435568	5.05	nonsynonymous	DNAJC10:NM_00127	0.44	0.003
99	**DNAJC10**	rs288334	chr2	183622547	183622547	T	G	hom	63.5	25	0.65	0.697247	n	nonsynonymous	DNAJC10:NM_00127	1	0
100	**FRZB**	rs7775	chr2	183699584	183699584	G	C	hom	60.5	35	0.11	0.154313	3.6	nonsynonymous	FRZB:NM_001463:e:	0.01	0.993
101	**ZNF804A**	rs4667001	chr2	185801747	185801747	G	A	hom	49	42	0.56	0.43029	3.22	nonsynonymous	ZNF804A:NM_19425	0.16	0.98
102	**ZNF804A**	rs12477430	chr2	185802363	185802363	A	G	hom	205	52	0.74	0.684635	2.05	nonsynonymous	ZNF804A:NM_19425	0.27	0
103	**FSIP2**	rs992822	chr2	186654592	186654592	A	G	hom	36	49	0.51	n	4.99	nonsynonymous	FSIP2:NM_173651:e:	1	n
104	**FSIP2**	rs10931199	chr2	186655726	186655726	G	A	hom	36	55	0.95	n	4.33	nonsynonymous	FSIP2:NM_173651:e:	0.69	n
105	**FSIP2**	rs17229201	chr2	186656956	186656956	T	C	hom	222	50	0.51	n	3.39	nonsynonymous	FSIP2:NM_173651:e:	1	n
106	**FSIP2**	rs10490391	chr2	186658438	186658438	C	T	hom	206	47	0.51	n	n	nonsynonymous	FSIP2:NM_173651:e:	0.19	n
107	**FSIP2**	rs10490392	chr2	186658565	186658565	G	T	hom	214	48	0.51	n	n	nonsynonymous	FSIP2:NM_173651:e:	0.52	n
108	**FSIP2**	rs2161036	chr2	186659359	186659359	G	T	hom	83	44	0.51	n	2.79	nonsynonymous	FSIP2:NM_173651:e:	1	n
109	**FSIP2**	rs10931200	chr2	186664963	186664963	C	A	hom	25	51	0.51	n	n	nonsynonymous	FSIP2:NM_173651:e:	1	n
110	**FSIP2**	rs36004074	chr2	186665432	186665432	G	A	hom	6.02	46	0.51	n	n	nonsynonymous	FSIP2:NM_173651:e:	0.88	n
111	**FSIP2**	rs11695215	chr2	186665824	186665824	A	G	hom	81.3	49	0.51	n	3.36	nonsynonymous	FSIP2:NM_173651:e:	0.04	n
112	**FSIP2**	rs7605884	chr2	186667121	186667121	T	C	hom	30	43	0.51	n	5.2	nonsynonymous	FSIP2:NM_173651:e:	0.74	n
113	**FSIP2**	rs7576532	chr2	186670104	186670104	A	C	hom	26.5	48	0.95	0.978639	2.78	nonsynonymous	FSIP2:NM_173651:e:	1	0.445
114	**FSIP2**	rs16827154	chr2	186670780	186670780	T	A	hom	5.29	55	0.48	0.512575	n	nonsynonymous	FSIP2:NM_173651:e:	0.09	0.005
115	**FSIP2**	rs4666689	chr2	186670979	186670979	T	C	hom	21.8	32	0.85	0.862876	n	nonsynonymous	FSIP2:NM_173651:e:	0.7	0.205381
116	**FSIP2**	rs17826534	chr2	186671357	186671357	G	A	hom	13.9	38	0.51	0.542319	3.07	nonsynonymous	FSIP2:NM_173651:e:	0.08	0.397062
117	**FSIP2**	rs1862066	chr2	186671912	186671912	G	A	hom	222	55	0.51	0.543956	3.65	nonsynonymous	FSIP2:NM_173651:e:	1	0.06
118	**ITGAV**	rs2230616	chr2	187532417	187532417	G	A	hom	213	44	0.99	0.99085	3.39	nonsynonymous	ITGAV:NM_002210:e	0.81	0.001
119	**CALCRL**	rs698577	chr2	188250301	188250301	T	A	hom	23	40	1	0.998531	n	nonsynonymous	CALCRL:NM_001271	1	0
120	**COL3A1**	rs1516446	chr2	189875421	189875421	T	G	hom	222	49	1	0.994925	n	nonsynonymous	COL3A1:NM_000090	1	0
121	**ANKAR**	rs1225090	chr2	190595258	190595258	G	T	hom	21	47	0.99	0.978471	n	nonsynonymous	ANKAR:NM_144708:	0.7	0.354752
122	**DNAH7**	rs13411834	chr2	196673533	196673533	A	G	hom	12.2	40	0.97	0.963252	5.06	nonsynonymous	DNAH7:NM_018897:(	1	0.332273
123	**GTF3C3**	rs11559078	chr2	197662526	197662526	T	C	hom	38	45	0.09	0.104029	n	nonsynonymous	GTF3C3:NM_001206	0.71	0
124	**CASP8**	rs3769823	chr2	202122995	202122995	A	G	hom	13	38	0.65	0.669471	2.78	nonsynonymous	CASP8:NM_0010801	0.76	0.373473
125	**ALS2CR12**	rs13014235	chr2	202215492	202215492	C	G	hom	96	24	0.65	0.646086	n	nonsynonymous	ALS2CR12:NM_0011	1	0
126	**TRAK2**	rs13022344	chr2	202264156	202264156	C	T	hom	222	48	0.53	0.571659	5.2	nonsynonymous	TRAK2:NM_015049:(	0.19	0.014
127	**ALS2CR11**	rs2714482	chr2	202357949	202357949	A	C	hom	30	41	1	n	2.1	nonsynonymous	ALS2CR11:NM_0011	0.74	n
128	**MPP4**	rs6725443	chr2	202557702	202557702	C	T	hom	62.5	25	0.98	0.972056	3.92	nonsynonymous	MPP4:NM_033066:e:	0.55	n
129	**NBEAL1**	rs4675323	chr2	204062061	204062061	A	G	hom	222	40	0.86	0.893814	3.88	nonsynonymous	NBEAL1:NM_001114	1	0.424196
130	**CYP20A1**	rs2043449	chr2	204116690	204116690	C	T	hom	171	37	0.88	0.888129	n	nonsynonymous	CYP20A1:NM_17753	0.48	0
131	**INO80D**	rs2909111	chr2	206911228	206911228	G	A	hom	191	42	0.37	0.31883	n	nonsynonymous	INO80D:NM_017759:	0.13	n
132	**METTL21A**	rs2551949	chr2	208477852	208477852	G	A	hom	36	42	0.89	0.834307	2.88	nonsynonymous	METTL21A:NM_0011	0.17	0.883
133	**C2orf80**	rs10804166	chr2	209036712	209036712	T	C	hom	41.5	31	0.87	0.890125	n	nonsynonymous	C2orf80:NM_001099:	0.1	0.036
134	**PIKFYVE**	rs10932258	chr2	209184980	209184980	G	A	hom	24	26	0.99	0.992151	5.83	nonsynonymous	PIKFYVE:NM_01504(	1	0
135	**PIKFYVE**	rs893254	chr2	209190519	209190519	A	T	hom	197	28	0.91	0.922344	4.24	nonsynonymous	PIKFYVE:NM_01504(	0.11	0.237
136	**PIKFYVE**	rs893253	chr2	209190528	209190528	C	G	hom	92	26	0.94	0.923112	n	nonsynonymous	PIKFYVE:NM_01504(	0.75	0
137	**PIKFYVE**	rs1529979	chr2	209191082	209191082	C	A	hom	13	40	0.94	0.922344	4.65	nonsynonymous	PIKFYVE:NM_01504(	1	0
138	**FN1**	rs1250209	chr2	216235089	216235089	C	T	hom	193	35	1	0.999846	5.6	nonsynonymous	FN1:NM_212476:exo	1	0
139	**USP37**	rs6436058	chr2	219319657	219319657	A	G	hom	110	42	0.98	0.989466	n	nonsynonymous	USP37:NM_020935:(	0	0.981
140	**CNPPD1**	rs4674361	chr2	220037393	220037393	A	G	hom	30	22	0.99	0.995617	2.79	nonsynonymous	CNPPD1:NM_01568(	0.38	0
141	**DNPEP**	rs907679	chr2	220250147	220250147	A	G	hom	138	22	1	0.999919	2.29	nonsynonymous	DNPEP:NM_012100:	1	0
142	**OBSL1**	rs1039898	chr2	220430203	220430203	C	T	hom	13.2	40	0.82	0.828228	2.12	nonsynonymous	OBSL1:NM_015311:(	1	n
143	**STK11IP**	rs681747	chr2	220462640	220462640	G	T	hom	19.8	22	0.86	0.836647	n	nonsynonymous	STK11IP:NM_052902	0	n
144	**STK11IP**	rs627530	chr2	220476443	220476443	C	T	hom	20	26	0.86	0.839759	n	nonsynonymous	STK11IP:NM_052902	0.01	n
145	**FARSB**	rs7185	chr2	223436607	223436607	C	T	hom	182	33	0.86	0.805859	4.33	nonsynonymous	FARSB:NM_005687:(	1	0
146	**COL4A3**	rs10178458	chr2	228111435	228111435	T	C	hom	23.8	25	0.81	0.770425	3.62	nonsynonymous	COL4A3:NM_000091	0.31	0.398642
147	**SP110**	rs3948464	chr2	231050715	231050715	A	G	hom	40	36	0.9	0.862679	n	nonsynonymous	SP110:NM_00118501	0.35	0
148	**SP110**	rs1365776	chr2	231072709	231072709	C	T	hom	39.3	43	0.79	0.702293	n	nonsynonymous	SP110:NM_00118501	0.16	0
149	**ECEL1**	rs1529874	chr2	233349588	233349588	G	A	hom	210	25	0.97	0.958019	4.88	nonsynonymous	ECEL1:NM_004826:(	1	0
150	**NGEF**	rs4973588	chr2	233834975	233834975	A	G	hom	222	51	0.88	0.869599	n	nonsynonymous	NGEF:NM_019850:e:	0.23	0.276
151	**INPP5D**	rs36127492	chr2	234066969	234066969	C	G	hom	200	33	0.72	0.569864	2.5	unknown	UNKNOWN	n	n
152	**COL6A3**	rs2270669	chr2	238243464	238243464	C	G	hom	13	24	0.8	0.835076	5.29	nonsynonymous	COL6A3:NM_004369	n	0.437509
153	**PASK**	rs1470414	chr2	242077496	242077496	C	T	hom	222	33	0.25	0.293403	n	nonsynonymous	PASK:NM_00125212	1	0.001
154	**HDLBP**	rs11891776	chr2	242203916	242203916	A	C	hom	144	31	1	n	5.64	nonsynonymous	HDLBP:NM_0012439	1	0
